# IoT-aware extreme machine learning for efficient health monitoring

**DOI:** 10.1186/s12913-026-14108-x

**Published:** 2026-04-30

**Authors:** Nithya Rekha Sivakumar

**Affiliations:** Department of Computer Sciences, College of Computer and Information Sciences, Princess Nourah bint Abdulrahman University (PNU), P.O. Box 84428, Riyadh, 11671 Saudi Arabia

**Keywords:** IoT health monitoring, Physiological and psychiatric parameters, Recursive extreme machine learning, Proximity projection pursuit, Slice Inverse Regression, Gaussian kernel function

## Abstract

Urban Health monitoring systems significantly improve public well-being. Internet technologies have enabled a new dimension of healthcare technology, generating a large amount of data for informed decision-making. Many researchers used machine learning-based classification because of their physical feature extraction. However, the accuracy level was not improved. The Kernel Slice Regressed Recursive Extreme Learning Machine (KSRRELM) Technique is developed for efficient healthcare monitoring with higher accuracy. The KSRRELM technique is used to identify health conditions of human well-being, including physiological and psychiatric parameters. Healthcare information is collected from patients at different locations. Recursive Extreme Learning Machine is used to analyze patients’ physical and mental health conditions. Collected patient information is considered input. Data preprocessing is carried out to handle missing values using proximity projection pursuit and outlier detection. kernel slice inverse Regression is used to analyze patients’ physiological and psychiatric parameters to identify whether the patient’s condition is normal or abnormal. The analyzed results are sent to the output layer. Efficient healthcare monitoring is carried out with higher accuracy. Observed results attained accuracy 95%, precision of 94%, recall of 97% and F1-score of 96%. KSRRELM technique realizes a reduction in error rate of 5%, time of 40 ms, and space complexity of 275 MB compared to traditional methods.

## Introduction

IoT-based health monitoring involves using interconnected devices to gather and transmit real-time health data for remote and continuous monitoring of individuals. This paradigm enhances patient care, detects health issues early, and improves overall healthcare efficiency. Physiological health monitoring involves continuously tracking vital signs such as heart rate, blood pressure, respiratory rate, and other biometric data. This real-time monitoring provides valuable insights into an individual’s overall health and allows for early intervention in case of abnormalities. Wearable and smart IoT devices have become instrumental in collecting and analyzing this data, enabling healthcare professionals to make informed decisions and adapt personalized treatment plans. Moreover, psychiatric health monitoring technology involves monitoring mental health indicators, such as stress levels, sleep patterns, and mood fluctuations. The ability to track these parameters over time provides an individual’s mental well-being, allowing for the timely identification of potential mental health disorders. Machine learning models play a vital role in making predictions and further enhancing the capabilities of health monitoring systems and treatment recommendations. A conventional CNN-based model provides insufficient accuracy. Machine-learning approach gives deficient precision. Optimization technique not achieve higher efficiency. In every existing methods unable to focus on computation cost minimization and time minimization. To concern these issues, a new technique called Kernel Slice Regressed Recursive Extreme Learning Machine (KSRRELM) Technique is developed for monitoring patients’ health conditions with higher accuracy with minimum time consumption.

### Problem definition

Health monitoring is a vital part to enhance the quality of humans. The evaluation of IoT in the medical field has emerged in monitoring the health activity of patients. Effectively monitoring and managing patients’ physiological and psychiatric health conditions are challenging. Many predictive models are developed that analyze continuous physiological data (e.g., heart rate, blood pressure) to identify patterns indicative of early-stage health issues. However, accurate predictions of patient health conditions were insufficient, failed to categorize huge amounts of data, health monitoring issues were not considered, and failed to handle missing values and outlier detection. In addition, timely monitoring of health-related issues requires real-time processing of physiological and psychiatric data streaming. Conventional machine learning models may face challenges in meeting time-efficient health monitoring. But it is challenging to analyze a huge memory. To address the issue, the proposed KSRRELM technique is introduced to enhance the accuracy and reduce time and space for handling health monitoring issues.

### Aim and objectives

This research aims to overcome the problems of accurate and timely health monitoring systems. The main objective is listed below. To monitor patients’ health conditions with maximum accuracy and minimal time, a novel KSRRELM technique has been developed, incorporating two key processes: preprocessing and classification. To handle missing data and detect outliers with lesser time and space complexity, the proximity projection pursuit imputation method and Ratcliff-Obershelp pattern matching are employed in the KSRRELM technique. To classify the patient as either in a normal state or an abnormal state with higher accuracy, Kernel Slice Inverse Regression is applied. To minimize the error, the damped least-squares method is employed to minimize the incorrect classification.

### Novelty and contribution

The novelty and contributions are illustrated as follows,

- For improving the accuracy and reducing time, the proposed KSRRELM is introduced for health monitoring systems.
- For investigating the patient’s physical and mental health conditions, the Recursive Extreme Learning Machine is employed with several layers, such as the input layer, hidden layers, and the output layer.
- Proximity projection pursuit imputation method and Ratcliff-Obershelp pattern matching are utilized to perform data preprocessing. Novel proximity estimation is used to measure the similarity among data points. The imputation technique is used to determine the possible missing values. The innovation of Ratcliff-Obershelp pattern matching is employed for confirming the data points and detecting outliers. In this way, time and space are reduced.
- Kernel Slice Inverse Regression is applied in the Recursive Extreme Learning Machine for executing data classification. The relationship between the input variables is estimated, and the patient’s normal state or an abnormal health issue state is investigated with higher accuracy. Also, the sum of squared error values is reduced with the innovation of the damped least-squares method. Classification of patient data is accurately obtained with less error.

### Organization of the paper

The rest of the paper is organized into various sections as follows. Section “Literature review” discusses the literature review and related terminologies. Section “Materials and methods” outlines the materials and methods used in the study. Section “Proposed methodology” details the system model and architecture of the proposed RGEOFML method. Section “Experimentation and analysis” presents experimentation and analysis, including various evaluation metrics. Section “Analysis sections” focuses on the analysis portion of the case study. Finally, Sect. “Conclusion” provides the conclusion and future scope.

## Literature review

### CNN based models

A CNN-based prediction model was designed by Gupta et al. [1] using IoT paradigms to predict health monitoring performance. However, the designed method failed to achieve higher accuracy when handling large amounts of patient data. A deep learning-based IoT-enabled real-time health monitoring system was introduced by Wu et al. [2], utilizing vital signs and extracting valuable information. However, it has a higher computational complexity. A pre-convoluted fast recurrent neural network (P-FRNN) was introduced by Jain et al. [3] to distinguish abnormal data related to the health of individuals with minimal time consumption. However, it failed to minimize the error rate in classification.

A novel machine-learning approach was designed by Rayan & Alanazi [4] for mental health monitoring. This approach considers various variables relevant to mental health, including behavioral traits such as exercise routines, sleep patterns, and social interactions, as well as psychological data. A smart healthcare system was developed by Islam et al. [5] for an IoT environment to monitor a patient’s basic health signs with minimal error rates. However, the complexity of the time was not minimized. The Machine Learning algorithms were developed by Talaat et al. [6], and correlation methods were used to improve the performance of stress monitoring. Patients using IoT medical devices were developed by Monteith et al. [7] to monitor general medical conditions. M/M/c/K queuing network model was employed by Silva et al. [8] for IoHT infrastructure with a three-layer cloud/fog/edge computing field. However, the accuracy level was not improved, and the time complexity was not reduced. An IoT-based real-time health monitoring system was developed by Bhuiyan et al. [9] to monitor and report people’s health conditions with minimal error rates. However, the time complexity of the health monitoring system was not minimized. CNN and LSTM weredesignedby KattaB [10] with higher improvement in disease detection for the Internet of Things. But the time was higher. A meta-learning-based personalization method was designed by Jia et al. [11] to perform patient-specific detection with higher detection accuracy. Cloud-dew architecture was introduced by Karmakar et al. [12] for health monitoring systems with bio signals. However, the machine learning-based hybrid intelligence was not applied to a decision-making system. A Convolutional Neural Network was developed by Banerjee et al. [13] for an efficient remote health monitoring approach. However, it failed to detect conditions such as depression levels. An IoT-based system was introduced by Islam et al. [14] for remote monitoring and early detection of health problems. However, deep learning models were developed to enhance accuracy further in classifying health problems. E-health technologies and remote patient monitoring were introduced by Singh et al. [15], but the mental health conditions of patients remained unaddressed. A deep learning architecture was developed by Mohana et al. [16] to achieve a higher rate of classifying the input instances. However, integrating multimodal signals for improved classification of instances remained unsolved. A multi-level deep neural network with a hierarchical approach was developed by Kumar et al. [17] for IoT-based mental stress state detection. A real-time health monitoring system was developed by Sangeetha Lakshmi et al. [18] based on IoT to monitor patients’ healthcare conditions. However, the numerous health monitoring data were not handled. A novel algorithm called the iCloud Assisted Intensive Deep Learning (iCAIDL) method was developed for healthcare monitoring by Kondaka et al. [19]. However, the higher accuracy was not achieved. A structural design and execution of healthcare patient monitoring was performed by Akkaş et al. [20] for a biomedical application.

The Mask-RCNN technique was developed by Ahmed et al. [21] to extract various parameters from the patient’s body to achieve a high true-positive rate and minimize the false-positive rate in health monitoring. Deep learning technology was introduced by Verma et al. [22] to enable automated monitoring and provide real-life healthcare systems with higher accuracy and minimal latency. A Hybrid Deep Learning model was developed by Dang et al. [23] for structural health monitoring through Feature combination. However, the model’s complexity was not effectively minimized. The analysis of the Internet of Medical Things (IoMT) integrated with home health monitoring was conducted by Rodrigues et al. [24]. However, a machine learning technique was not employed to enhance the performance of the health monitoring system.

A Smart Health Monitoring System was introduced by Sheela et al. [25], employing a machine learning system to monitor patients remotely. A Linear Discriminant Analysis (LDA) was developed by Dahan et al. [26] to select the optimal features for predicting abnormal or normal data. A novel deep learning-based system was developed by Rachakonda et al. [27] to monitor an individual’s stress levels through human body temperature and motion during physical activity. As developed by Zamani et al. [28], a novel deep learning model is designed for a wearable-enabled smart health monitoring system to detect sleep quality by collecting data during sleep activity. However, the overall monitoring system performance was not improved. Deep Learning and Machine learning algorithms were introduced by Sujith et al. [29] to analyze health data for the early detection of diseases. An integrated wearable monitoring system was introduced by Li et al. [30] and utilizes artificial intelligence (AI) for the diagnosis of respiratory abnormalities. A smart real-time health monitoring system was developed by Albahri et al. [31] for hospitals’ dispensers using a health recommender framework. The analysis of deterministic errors, stochastic errors, accuracy, and energy efficiency has remained unresolved. A novel traceable patient health data search method was introduced by Zhou et al. [32] for hospital management, aiming to recognize patients with particular features from the health monitoring data. An IoT-based smart instrumentation system was developed by Chowdhry et al. [33] to analyze the health structure. However, the latency of this IoT-based instrumentation was not reduced.

The Dynamic Multi-Layer Perceptron (DMLP) was developed by Sirisha et al. [34] to determine fetal health with higher accuracy. But the time was not considered. The study focused on ten distinct machine learning methods by Akhund & Al-Nuwaiser [35] for forecasting cardiovascular disease. However, the recall was not improved. The Monte Carlo-based method was utilized by Jabeen et al. [36] for observing patients’ health. The real-time health status was determined by random selection. It offers a real-time monitoring system for restrictions ontheextent of viral disease. Nevertheless, it failed to minimize the time. CNN and LSTM were developed by Alharbe & Almalki [37] to improve healthcare diagnosis. However, it failed to find the early disease. An ensemble deep learning model was employed by Gayathri et al. [38] for discovering health problem diagnosis. But the accuracy was not improved. The study focused on deep learning-based structural health monitoring by Cha et al. [39] with higher reliability. An IoT-based secure health monitoring system was introduced by Qi [40] for physical conditions to handle patients’ health issues. However, it failed to find the patient’s condition as normal or abnormal.

### Optimization based models

A Hybrid Dingo Coyote Optimization with Deep Ensemble Learning (HDCO-DEL) was developed by Abidi et al. [41] for Smart Health Monitoring with big data. While the method improves overall high accuracy, it does not address the minimization of space complexity.

### Hybrid models

A logistic regression-based classifier was developed by Iqbal et al. [42] to determine the stress classification accuracy of the model, but it did not consider physical activity. Existing methods of technical gaps are illustrated in Table 1.

**Table 1:** Technical gaps in existing techniques

Methods/Technique	Technical Aspect	Observed gap
Convolutional neural network (CNN)	Predict health monitoring performance	Lack of improvement in accuracy when handling large amounts of patient data.
A deep learning-based IoT-enabled real-time health monitoring system	Extracting vital signs and valuable information from the patient	It has a higher computational complexity
A Hybrid Dingo Coyote Optimization with Deep Ensemble Learning (HDCO-DEL)	Improves the overall high accuracy of Smart Health Monitoring with big data	Lack of reducing the space complexity
Pre-convoluted fast recurrent neural network (P-FRNN)	Distinguishing the abnormal health of individuals with minimal time consumption	Lack of minimizes the error rate

### Related terminologies

Integrating Internet of Things (IoT) technologies is pivotal in efficient health monitoring. This involves seamless connectivity of various devices to collect patient data for the health monitoring ecosystem.

**Technical Highlights:**

- Preprocessing: Implementing a technique for collecting the data and involving the cleaning and transformation of raw data into a format suitable for further processing.
- Predictive analysis: Implementing extreme learning for predictive analytics to monitor patient health performance, with trade-offs between accuracy and error rate.

## Materials and methods

### Materials

To experiment, Lifespans Fitbit dataset and MIMIC Dataset are obtained from ref [43] and ref [44] to determine patients’ health conditions. The data was collected from 71 participants and included physiological and psychiatric data, such as age, gender, user ID, temperature, oxygen saturation (SpO2), heart rate, beats per minute, calories, sleep duration, BMI, stress score, etc. Based on these parameters, normal and abnormal patient health conditions are detected.

### Methods

The kernel Slice Regressed Recursive Extreme Learning Machine (KSRRELM) Technique is introduced for efficient healthcare monitoring with higher accuracy and minimal time consumption.

## Proposed methodology

In this paper, the KSRRELM technique is developed to automatically monitor patients’ physiological and psychiatric health. IoT has been applied in many real-time applications, and the main aim is to enhance this technology in the current healthcare system.

### System model

In this model, IOMT devices ${d_i} = {d_1},{d_2},{d_3}, \ldots .,{d_m}$ are positioned in the patient’s body to monitor the patient’s physiological and psychiatric data samples or training samples ${X_1}, {X_2}, {X_3} \ldots {X_m}$ and perform data preprocessing. Then, the relationship between the data samples is determined for patient health prediction.

### Architecture of proposed methodology

The architecture of the proposed KSRRELM technique refers to its underlying structure, design, or outlines. The architecture plays a crucial role in determining its functionality, efficiency, and effectiveness.

**Fig. 1:**
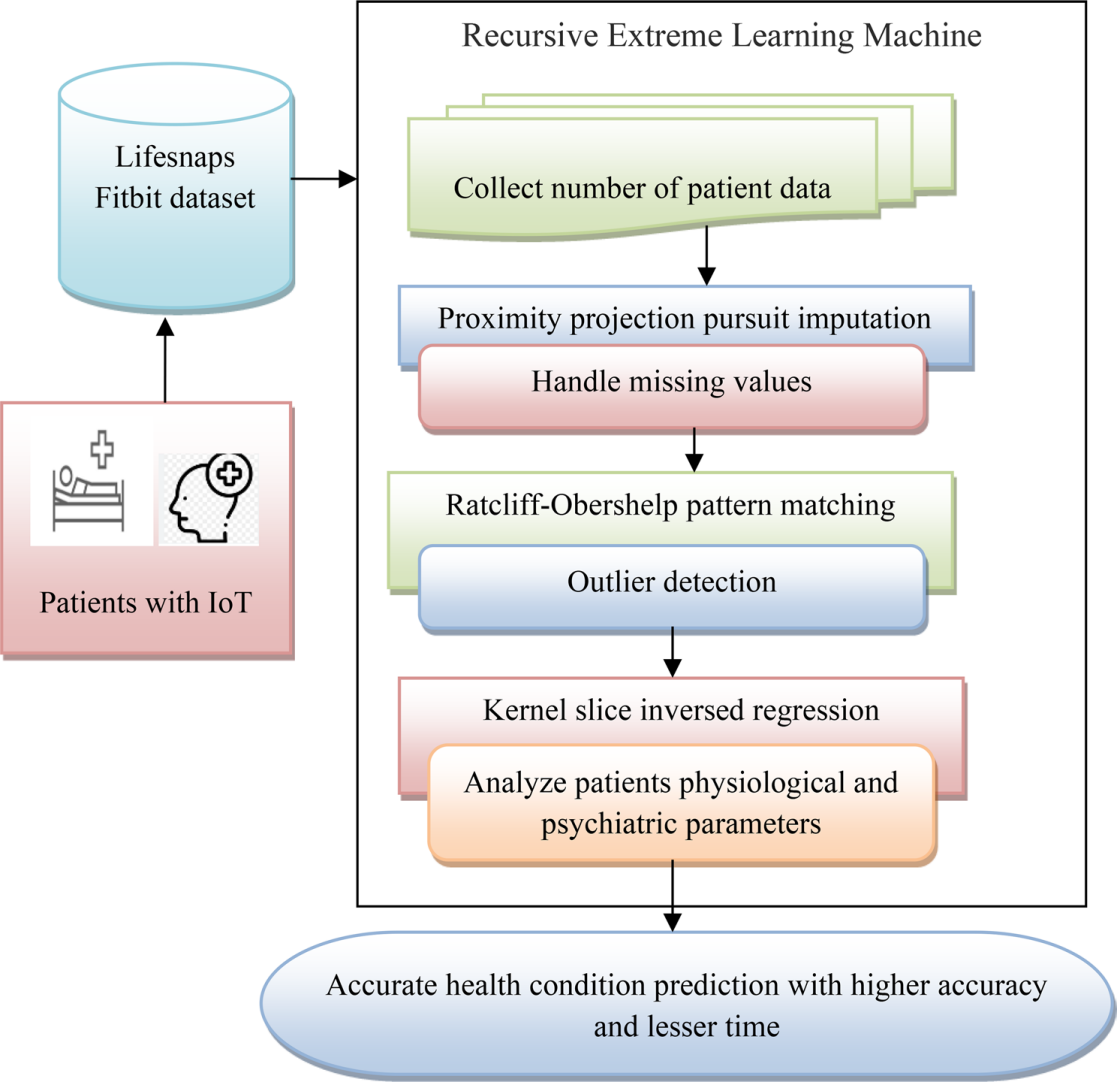
Architecture of the proposed KSRRELM technique

Figure 1 shows the architecture of the proposed KSRRELM technique for Physiological and Psychiatric monitoring in healthcare IoT.

### Data acquisition phase

The data acquisition phase is vital in collecting and gathering relevant data from the dataset for further analysis in machine learning. This phase involves systematically obtaining raw data from sources to support decision-making or problem-solving. In this phase, data are collected from the wearable IoT device, which collects physiological and Psychiatric data. The physiological data are heart rate, temperature, oxygen saturation, etc., and the psychiatric data contain various variables relevant to mental health. These variables include behavioral traits like exercise routines, sleep patterns, and social interactions, as well as psychological traits like mood, stress levels, and emotional states. IoT devices connect with patients in healthcare services. These devices are attached to the patient’s body to collect healthcare information.

### Recursive Extreme Learning Machine

**Fig. 2:**
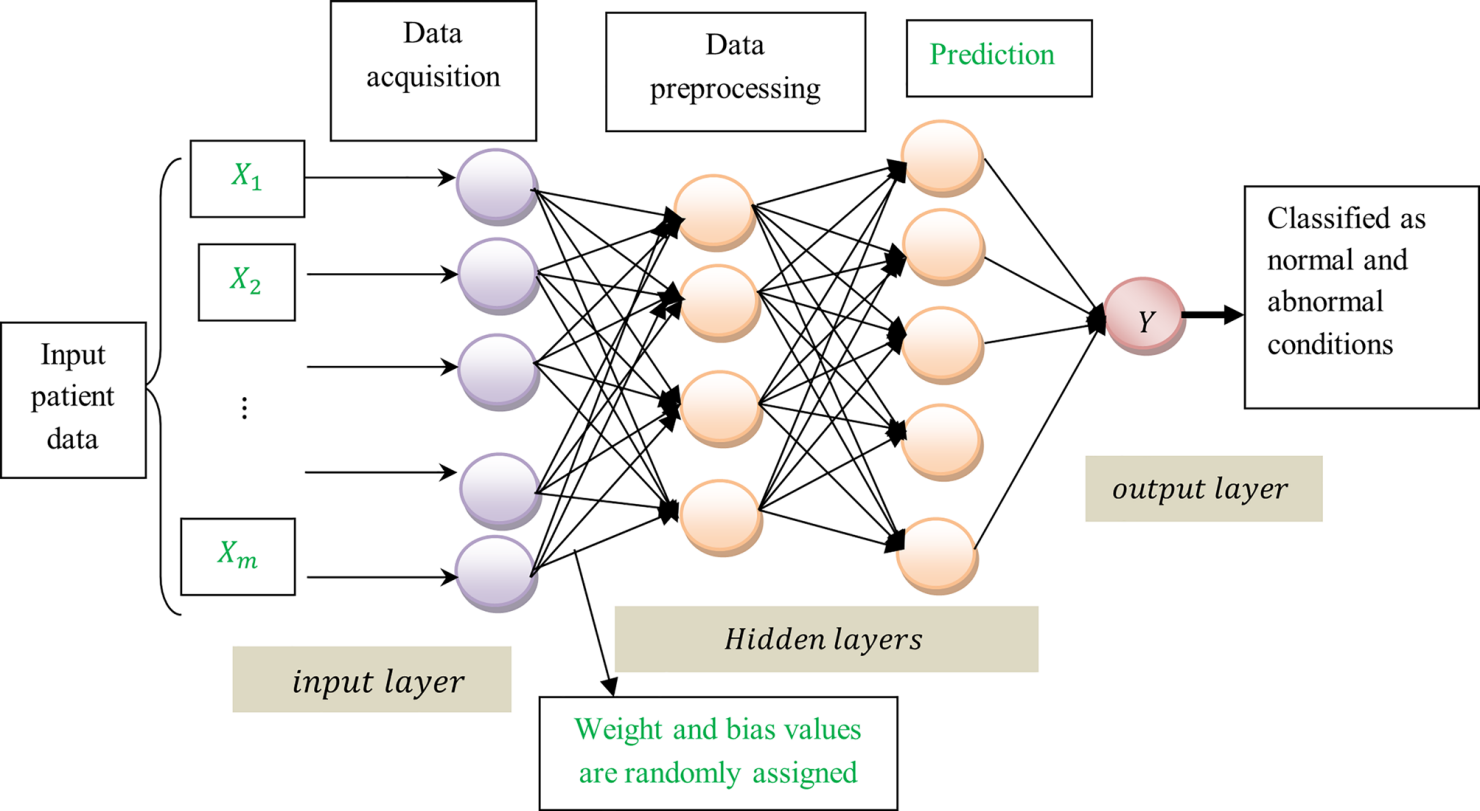
Architecture diagram of the Recursive Extreme Learning Machine

The next phase of the proposed KSRRELM technique is the patient data analysis using a Recursive Extreme Learning Machine. Recursive Extreme Learning Machine (ELM) is a machine learning algorithm that belongs to the family of single-hidden-layer feed-forward neural networks. It is used to analyze the patient’s physical and mental health conditions with the help of three layers: one input layer, two hidden layers, and one output layer. The analysis process gets repeated at different time intervals, termed recursion. The architecture diagram of the Recursive Extreme Learning Machine is shown on Fig. 2.

Figure 2 illustrates the architecture diagramof the Recursive Extreme Learning Machine, which includes the input layer, hidden layer, and output layer. Let us consider the training set $\left\{ {{X_i}, Y} \right\},$ where ‘${X_i}$’ denotes input patient data training samples, ${X_2}, {X_3} \ldots {X_m}$, and ‘$Y$’ represents the corresponding output label, indicating the category to which it belongs. Each layer comprises neurons used for transferring information from one layer to another. The above network structure includes the input layer and multiple hidden layers, and the output layer. The input layer consists of the input of the number of patient data from the dataset in data acquisition. The input is transformed into the first hidden layer. In the first hidden layer, preprocessing is used to handle missing values and find outliers. It sends to the second hidden layer. Followed by the Kernel Slice Inversed Regression is performed at the second hidden layer to predict the patient’s condition. Finally, the value is sent to the output layer, where damped least-squares are applied to obtain the accurate classification results.

The activity of the neuron at the input layer is measured. The neuron parameters include weights and biases.

1 $$Q = \mathop \sum \limits_{i = 1}^m [{X_i}* {\delta _{ih}}] + w$$

Equation (1), the input of neurons ‘$Q$’, ${\delta _{ih}}$ denotes a weight between the input layer neuron and the hidden layer neuron, $w$ denotes a bias, ${X_i}$ denotes input patient data or training samples. Then, the input is transferred into the hidden layer of the neurons. In that layer, data pre-processing is carried out to minimize time complexity and increase patient health condition prediction accuracy.

#### Data processing

Data pre-processing is a fundamental step in patient data analysis that involves cleaning, transforming, and organizing raw data into a suitable format. A proximity projection pursuit imputation method is proposed for handling missing values. This imputation technique is a statistical method that finds the possible missing values at each iteration for particular features through conditional statistical means.

To start with, the raw input dataset and formulated in the form of a matrix. $X$’, concerning rows and columns as given below.

2 $$X = \left[ {\matrix{ {{X_{11}}} & {{X_{12}}} & \ldots & {{X_{1n}}} \cr {{X_{21}}} & {{X_{22}}} & \ldots & {{X_{2n}}} \cr \vdots & \vdots & \ldots & { \vdots } \cr {{X_{m1}}} & {{X_{m2}}} & \ldots & {{X_{mn}}} \cr } } \right]$$

The input vector formulation is given in Eq. (2), where the column indicates an ‘n’ number of features or attributes, and the ‘$m$’ row indicates the number of patent data. The conditional statistical mean formula is estimated as given below,

3 $$M = {{\sum {{X_i}} } \over m}$$

4 $$\sum {{X_i}} = {X_1} + {X_2} + {X_3} + \ldots + {X_m}$$

Equations (3) and (4), $M$ denotes a conditional statistical mean, $\sum {{X_i}}$ indicates a sum of all the data values with the respective column, $m$ denotes the total number of values in the particular column. Then, the mean value is imputed to the corresponding missing cell in the dataset. After that, proximity estimation often involves defining similarity measures between data points. Common measures include distance,

5 $$P = \min D$$

6 $$D = \left| {{X_{ij}} - {X_m}} \right|$$

Equations (5) and (6), $P$ denotes an outcome of proximity estimation, ${X_{ij}}$ denotes a neighboring data point, ${X_m}$ denotes imputed data points, $D$ indicates a deviation between the data points. The imputed missing value should exhibit minimal deviation ‘$\min D$’ from their neighboring value. Therefore, the proposed technique accurately handles the missing value.

Followed by outlier detection is performed in the preprocessing phase. It is a process used in data analysis to identify points that deviate significantly from most of the dataset. Outliers, also known as variance it make errors in the data. Therefore, detecting outliers is fundamental for maintaining data quality. Ratcliff-Obers’ help’s pattern matching method is applied to verify the data points. The mathematical formula for computing the matching is given below,

7 $$ R = 2*{{{X_i}\mathop \cap \nolimits^ {X_j}} \over {\left| {{X_i}} \right| + \left| {{X_j}} \right|}}$$

8 $$MD = \left| {{X_i}\mathop \cap \nolimits^ {X_j}} \right|$$

Equations (7) and (8), $$ R$$ denotes a matching coefficient, $${X_i}$$ and $${X_j}$$ represents one data point, $$MD$$ denotes a mutual dependence between the data points. $$\left|{{X_i}} \right|$$ and $$\left| {{X_j}} \right|$$ denotes the set’s cardinality and several terms in the given sets. The coefficient provides the pattern-matching results between 0 and 1.

9 $$Z = \left\{ {\matrix{ {NDP, R > 0.5} \cr {ODP, R < 0.5} \cr } } \right.$$

Equation (9), $Z$ denotes an output function, $NDP$ denotes a normal data point. If the coefficient is greater than 0.5, then the data point is classified as normal, $ODP$ denotes an outlier data point when the coefficient value is less than 0.5. This method of preprocessing minimizes the time complexity and error prediction.

#### Classification using Kernel Slice Inversed Regression for patient health monitoring

The preprocessed data points are transferred into a second hidden layer of extreme learning for patient health monitoring. Kernel Slice Inversed Regression combined with slice inverse regression and Kernel trick. Data is mapped into a higher-dimensional feature space using a Gaussian kernel function. Slice inverse regression is a dimension reduction technique that aims to discover a lower-dimensional subspace that captures the essential information for predicting a response variable. It works by dividing the range of the response variable into slices and then examining the conditional distributions of the predictors within each slice. Kernel Slice Inversed Regression identifies linear relationships between the response and input variables. The response variable or output variables are represented as normal state or abnormal state and its input variables (i.e., physiological and psychiatric parameters). The regression algorithm involves slicing the input data into different directions (i.e., slices) and performing these steps on each slice to find the most informative directions for dimensionality reduction. Kernel Sliced Inverse Regression is a method used for dimension reduction. It’s applied to physiological (e.g., heart rate, sleep patterns) and psychiatric (e.g., anxiety levels, stress) parameters to identify health conditions and reduce the complexity of the data. Kernel Sliced Inverse Regression focuses on preserving the information relevant to the relationship between the predictor variables and the outcome (e.g., disease diagnosis, treatment response).

Let us consider the response variable $Y$ and an input vector $X$.

10 $$Y = f \left({ {\beta _1}X, {\beta _2}X, \ldots {\beta _n} X} \right)$$

Equation (10), $$\beta $$ denotes a regression coefficient, $$X$$ denotes a vector of input variables, $$f $$ denotes a function relating the linear combinations of the input variables to the response variable $$Y$$. The regression coefficients represent the relationship between the variables. The kernel function measures the relationship between the input variables to analyze the data.

11 $$ K \left({ {X_i},{X_j}} \right) = exp \left({0.5*{{\mathop \sum \nolimits_{i = 1}^n {{\left[ {{X_i} - {X_j}} \right]}^2}} \over {{\sigma ^2}}}} \right)$$

Equation (11), $K$ denotes a Gaussian kernel function that measures the relationship between the data ${X_i}$ and ${X_j}$, $\sigma$ denotes the deviation between the data. The kernel provides the outcomes ranging from 0 to 1. This analysis process gets repeated at different time intervals, which is termed recursion. The output of the hidden layer is expressed as follows.

12 $$H = \mathop \sum \limits_{i = 1}^L A \left({ {\delta _{ho}} K + w} \right) $$

Equation (12), $H$’ represents the hidden layer output, $A$ indicates a Sigmoid activation function, ‘${\delta _{ho}}$’ denotes the weight between the hidden layer neurons, $K$ represents the Gaussian kernel function.

13 $$A = {1 \over {1 + \exp \left({K \left( { {X_i},{X_j}} \right)} \right)}}$$

Equation (13), To minimize error, the damped least-squares method is used to minimize a sum of squared error values.

14 $$ DF = \arg min{\left[ {Y - Y{^{\prime}}} \right]^2}$$

Equation (14), ‘$DF$ denotes an output of the damped least-squares method, $\arg min$ indicates an argument of minimum function, $Y$ denotes an expected outcome and $Y{^{\prime}}$ represents actual classification results. Accurate classification results are achieved at the output layer by minimizing the error function. This approach ensures the correct classification of patients with normal and abnormal conditions based on their physiological and psychiatric data. The algorithmic process kernel Slice Regressed Recursive Extreme Learning Machine (KSRRELM) is given below,


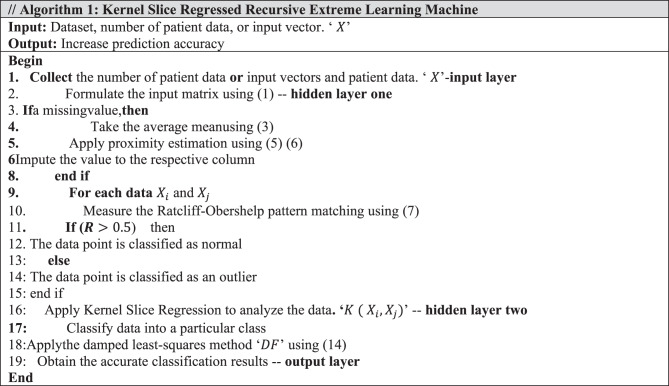


Algorithm 1, as presented above, outlines the steps involved in the Kernel Slice Regressed Recursive Extreme Learning Machine for healthcare patient monitoring. Initially, the algorithm collects physiological and psychiatric data from the dataset, feeds it into the input layer. Following data collection, preprocessing is carried out to organize the dataset in the first hidden layer. The algorithm calculates the patient data using the preprocessed outcomes by employing kernel Slice Inverse Regression. The analysis enables the accurate classification of patient data. Consequently, the classification results serve as a basis for monitoring patients’ health conditions.

## Experimentation and analysis

### Experimental setup

In this section, the KSRRELM technique, along with two existing methods, CNN [1] and a deep learning-based IoT-enabled real-time health monitoring system [2], HDCO-DEL [41], P-FRNN [4], and DMLP [36], are implemented using Python. The evaluation is performed using the Lifespans Fitbit dataset, obtained from ref [43], and the MIMIC dataset attained from ref [44]. The CSV file consists of six columns. The primary objective is to determine the health conditions of patients. This dataset is multi-modal, longitudinal, and geographically distributed. The data was collected from 71 participants, used for real-time ecological momentary assessments, as well as a Fitbit Sense smartwatch, and they consented to making these data available for psychology and behavioral sciences. The dataset includes physiological and psychiatric data, such as age, gender, user ID, temperature, oxygen saturation (SpO2), heart rate, beats per minute, calories, sleep duration, BMI, stress score, etc. Based on these parameters, normal and abnormal patient health conditions are detected. The experiments are conducted using the “csv_rais_anonymized/daily_fitbit_sema_df_unprocessed.csv” file from the dataset to detect patient health conditions. The proposed KSRRELM technique’s objective is to enhance accuracy, precision, recall, and F1-score, and minimize the time and space. Based on the objective, the existing methods, such as the deep learning-based IoT-enabled real-time health monitoring system [2], HDCO-DEL [41], P-FRNN [4], and DMLP [36] are taken as base papers. These two base papers are explained to understand the proposed technique. The proposed technique concept is derived by considering the problems of these base papers. The drawbacks of these methods are effectively addressed by implementing the proposed technique.

### Experimental parameters

This section presents a comparative analysis of the KSRRELM technique and existing methods, CNN [1], and a deep learning-based IoT-enabled real-time health monitoring system [2], HDCO-DEL [41], P-FRNN [4], and DMLP [36]. Based on the objective of the proposed KSRRELM technique, experimental parameters such as accuracy, precision, recall, F1-score, AUC, time, and space complexity are selected for experimental purposes. In our work, the Proximity projection pursuit imputation method and Ratcliff-Obers help pattern matching are used to handle missing values and detect outliers with lesser time and space complexity. Next, the Kernel Slice Inverse Regression is used to classify the normal and abnormal conditions of patients. This aids in improving the accuracy, precision, recall, and F1-score. The performance of each technique in terms of these metrics is illustrated through a table and graphical representations. In below Abbreviations, illustrate the briefly explained in all notations.

**Accuracy** is defined as the number of patient data or data samples accurately classified into normal or abnormal conditions. The overall accuracy rate is measured as follows:

15 $$ACC = \sum\limits_{i = 1}^m {\left[ {{\matrix{Number\;of\;{\mkern 1mu} data{\mkern 1mu} {\mkern 1mu} \hfill \cr \quad accurately\;classified \hfill \cr}\over {{X_i}}}} \right]} *100$$

Equation (15), $ACC$ indicates accuracy, ${X_i}$ denotes the number of data samples, $m$ denotes the total number of data samples. Therefore, accuracy is measured in percentage (%).

**The error rate** is defined as the number of patient data or data samples incorrectly classified into normal or abnormal conditions. The overall accuracy rate is measured as follows:

16 $$ERE = \sum\limits_{i = 1}^m {\left[ {{\matrix{Number{\mkern 1mu} {\mkern 1mu} of{\mkern 1mu} {\mkern 1mu} data{\mkern 1mu} \hfill \cr \quad {\mkern 1mu} incorrectly{\mkern 1mu} {\mkern 1mu} classified \hfill \cr} \over {{X_i}}}} \right]} *100$$

Equation (16), $ERE$ indicates error rate, ${X_i}$ denotes the number of data samples, $m$ denotes the total number of data samples. Therefore, the error rate is measured in percentage (%).

**Time complexity:** The time consumed by the algorithm to classify the patient’s health conditions. It is mathematically calculated as follows,

17 $$TCY = \mathop \sum \limits_{i = 1}^m {X_i}*time \left( {CDS} \right)$$

Equation (17), $TCY$ indicates a time complexity, $m$ denotes the number of patient data or data samples, $time \left( {CDS} \right)$ represents the time for classifying the single data sample. The time complexity is measured in milliseconds (ms).

**Space complexity:** The memory space the algorithm consumes to classify the patient’s health conditions. It is mathematically calculated as follows,

18 $$SCM = \mathop \sum \limits_{i = 1}^m {X_i}*MS \left( {CDS} \right)$$

Equation (18), $$SCM $$ indicates a space complexity, $$m$$ denotes the number of patient data or data samples, $$MS \left( {CDS} \right)$$ represents a memory space for classifying single-data samples. The space complexity is measured in Kilobytes (KB).

**Precision:** The precision is measured based on the number of true positives and false positives. Therefore, it is calculated as,

19 $$Pre = \left[ {{{{T_p}} \over {{T_p} + {F_p}}}} \right]$$

Equation (19), $Pre$ indicates a precision, ${T_p}$ indicates a true positive, ${F_p}$ denotes a false positive.

**Recall or False positive rate:** It is defined as the ratio between the number of true positive rates accurately classified as positive to the total number of positive samples. A higher recall value indicates that the model is better at identifying positive samples. It is formulated as

20 $${R_C} = \left[ {{{{T_p}} \over {{T_p} + {F_n}}}} \right]$$

Equation (20), ${R_C}$ indicates a recall, ${T_p}$ indicates a true positive, ${F_n}$ denotes a false negative.

**F1-Score:** It is measured as the average value of both precision as well as recall. It is computed as follows,

21 $$ F = \left[ {2{\bf{\it{*}}}{{Pre *{R_C}} \over {Pre + {R_C}}}} \right]$$

Equation (21), ‘$F$’ represent the F1-Score, ‘$Pre$’ indicates precision and ‘${R_C}$’ denotes recall.

**AUC:** AUC (Area under the Curve) is a characteristic employed to calculate the performance of binary classification models. True Positive Rate ‘$TPR$’ is also called recall. False Positive Rate (FPR) is the ratio of wrongly predicted positive observations to the actual negatives.

22 $$FPR = {{{F_p}} \over {{F_p} + {T_n}}}$$

Equation (22), ‘${F_p}$’ indicates false positive results, ‘${T_n}$’ refers to true negative. Therefore, the ROC curve is plotted with TPR on the y-axis and FPR on the x-axis.

Table 2 provides a graphical representation of the accuracy of classification diagnosis using the Lifesnaps Fitbit dataset concerning the number of data samples ranging from 500 to 5000. The table results indicate that the proposed KSRRELM technique achieves higher accuracy compared to existing methods [1, 2, 41, 4, 36], respectively. The data samples used for the experiment range from 500 to 5000. For each method, ten different runs are carried out with varying numbers of data samples. The observed results demonstrate that the proposed KSRRELM technique model performs better than conventional deep learning methods. The overall performance of the accuracy using the KSRRELM technique is improved by 5, 8,11, 13 and 3% when compared to [1, 2, 43, 4, 36], respectively. This is because of applying recursive extreme learning machines to analyze patient physiological and psychiatric data with kernel Sliced Regression in the hidden layer. Based on the analysis, the KSRRELM technique successfully classifies the normal and abnormal conditions of the patients.

**Table 2:** Comparison of accuracy using Lifesnaps Fitbit dataset

Number of data samples	Accuracy (%)					
	KSRRELM	CNN	Deep learning-based IoT-enabled real-time health monitoring system	HDCO-DEL	P-FRNN	DMLP
500	95.2	92.4	90	88.4	86.4	93.6
1000	94.5	91.2	89.8	87.5	85.5	93
1500	94.73	91.06	89.66	87	85.66	93.2
2000	96.8	92.75	88.25	86	84.25	94.5
2500	94.64	91.4	89.4	86.2	84	92.88
3000	95.16	89.5	87.06	85.16	83.16	92.5
3500	95.57	89.2	86.05	84.42	82.14	91.42
4000	96.37	90.65	88.07	86.37	83.9	92.87
4500	95.88	90.22	87.22	85.66	83.44	93.33
5000	94.9	90	88.04	85.1	83.7	92

Table 3 provides a graphical representation of the accuracy of classification diagnosis using the MIMIC dataset concerning the number of data samples ranging from 500 to 5000. The table results indicate that the proposed KSRRELM technique achieves higher accuracy compared to existing methods [1, 2, 41, 4, 36], respectively. The data samples used for the experiment range from 500 to 5000. For each method, ten different runs are carried out with varying numbers of data samples. The observed results demonstrate that the proposed KSRRELM technique model performs better than conventional deep learning methods. The overall performance of the accuracy using the KSRRELM technique is improved by 5, 8,10, 13 and 2% when compared to [1, 2, 43, 4, 36], respectively. This is due to Kernel Slice Inverse Regression is used to classify the normal and abnormal conditions of patients which aims to improve the accuracy.

**Table 3:** Comparison of accuracy using MIMIC dataset

Number of data samples	Accuracy (%)					
	KSRRELM	CNN	Deep learning-based IoT-enabled real-time health monitoring system	HDCO-DEL	P-FRNN	DMLP
500	96.2	93.4	91	89.4	87.4	94.6
1000	95.5	92.2	90.8	88.5	86.5	94
1500	95.73	92.06	90.66	88	84.66	94.2
2000	97.8	93.75	89.25	87	85.25	95.5
2500	95.64	92.4	90.4	87.2	85	93.88
3000	96.16	90.5	88.06	86.16	84.16	93.5
3500	96.57	90.2	87.05	85.42	83.14	92.42
4000	97.37	91.65	89.07	87.37	84.9	93.87
4500	96.88	91.22	88.22	86.66	84.44	94.33
5000	95.9	91	89.04	87.1	85.7	94

**Fig. 3:**
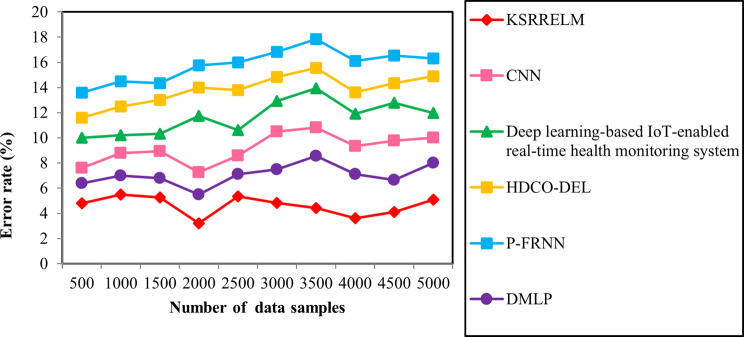
Graphical representation of the error rate for Lifesnaps Fitbit dataset

Figure 3 illustrates the graphical representation of the error rate diagnosis using the Lifesnaps Fitbit dataset versus the number of data samples taken from the dataset. The graphical results indicate that the proposed KSRRELM technique exhibits minimal error rate performance in classifying data samples compared to conventional deep learning methods. In the first iteration of experiments, 500 data samples were considered to compute the error rate. Different performance outcomes of the KSRRELM technique were also observed, and the results were compared. The overall comparison results suggest that the error rate of the KSRRELM technique was reduced by 49, 60, 66, 70 and 34% compared to existing methods. This is because of using damped least-squares method, which aims to minimize the sum of squared error values in data classification.

**Fig. 4:**
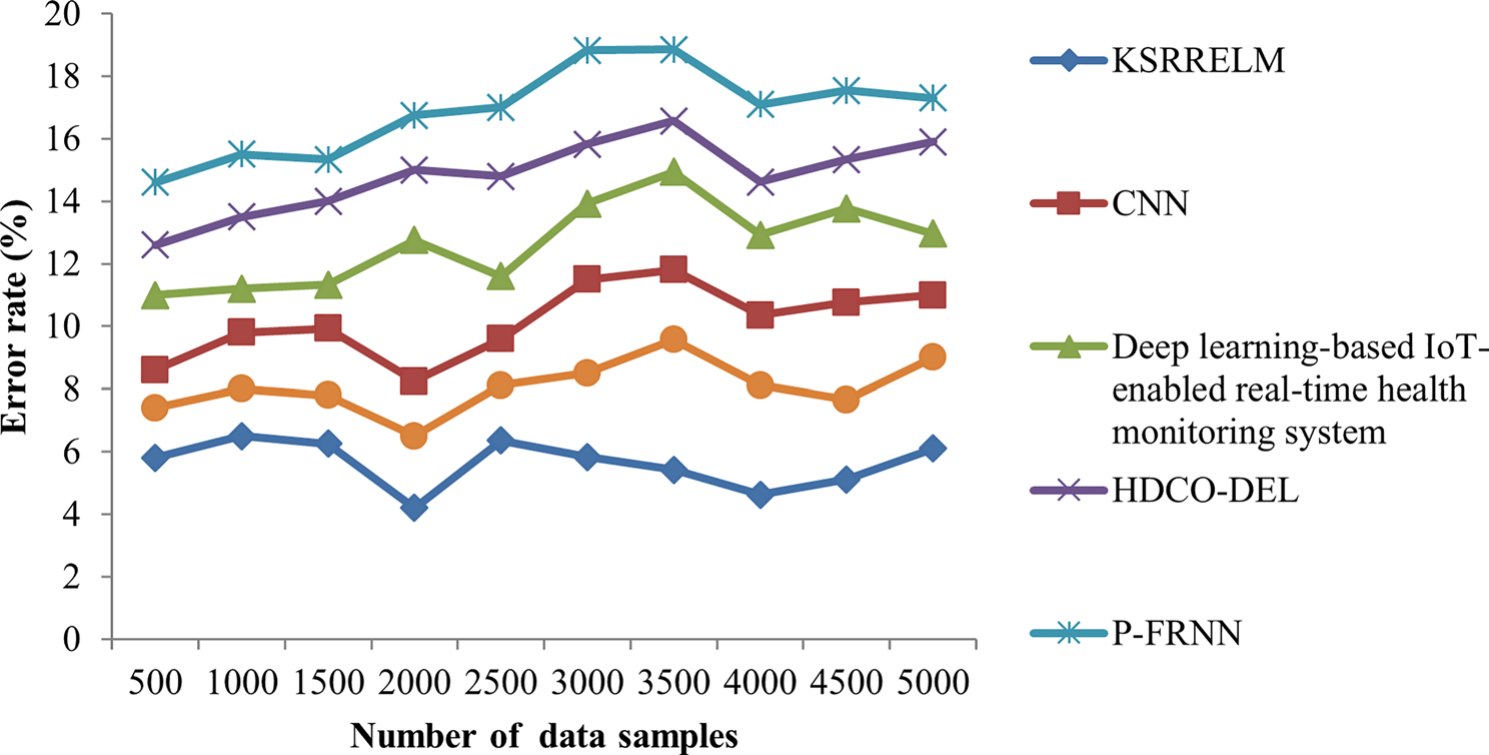
Graphical representation of the error rate for MIMIC dataset

Figure 4 illustrates the graphical representation of the error rate using the MIMIC dataset versus the number of data samples taken from the dataset. The graphical results indicate that the proposed KSRRELM technique exhibits minimal error rate performance in classifying data samples compared to existing deep learning methods. In the first iteration of experiments, 500 data samples were considered to compute the error rate. Different performance outcomes of the KSRRELM technique were also observed, and the results were compared. The overall comparison results suggest that the error rate of the KSRRELM technique was reduced by 44, 55, 62, 66 and 30% compared to existing methods. This is due to using outlier detection for minimizing the error rate.

Table 4 above depicts the time complexity of data classification using five different methods for Lifesnaps Fitbit dataset. In other words, the overall time consumption increases with the number of data samples. However, the KSRRELM techniques minimized the time complexity of data classification compared to existing deep learning techniques. The overall result specifies that the time complexity of KSRRELM techniques is significantly minimized by 6, 11, 15, 19 and 24% compared to existing methods. This is because the KSRRELM technique performs data preprocessing. In the data preprocessing step, missing data is handled by applying the proximity projection pursuit imputation method. This helps to fill in the missing value based on the average of all the data points in the particular feature. In addition, Ratcliff-Obershelp pattern matching is applied to verify the data points as normal or outliers. This process enhances data classification and efficiently minimizes time complexity.

**Table 4:** Comparison of time complexity using Lifesnaps Fitbit dataset

Number of data samples	Time complexity (ms)					
	KSRRELM	CNN	Deep learning-based IoT-enabled real-time health monitoring system	HDCO-DEL	P-FRNN	DMLP
500	25	27.5	30	32.5	35	37.6
1000	28	30	33	35	37	41
1500	33	36.75	39	42	45	47.25
2000	36.4	40	42	44	46	48
2500	38.75	41.25	43.75	45	47.5	52.5
3000	42.6	44.4	46.5	48	51	54
3500	46.2	48.3	50.75	52.5	54.25	57.75
4000	48.8	50	52	54	56	60
4500	51.75	53.1	55.8	58.5	60.75	62.55
5000	54	56.5	60	62.5	65	69

Table 5 above depicts the time complexity of data classification using the MIMIC dataset with five different methods. In other words, the overall time consumption increases with the number of data samples. However, the KSRRELM techniques minimized the time complexity of data classification compared to existing deep learning techniques. The overall result specifies that the time complexity of KSRRELM techniques is significantly minimized by 6,12, 15, 20 and 24% compared to existing methods. This is due to utilizing Proximity projection pursuit imputation method for reducing the time complexity.

**Table 5:** Comparison of time complexity using MIMIC dataset

Number of data samples	Time complexity (ms)					
	KSRRELM	CNN	Deep learning-based IoT-enabled real-time health monitoring system	HDCO-DEL	P-FRNN	DMLP
500	26	28.5	31	33.5	36	38.6
1000	29	31	34	36	38	42
1500	34	37.75	40	43	46	48.25
2000	37.4	42	43	45	47	49
2500	39.75	42.25	44.75	46	48.5	53.5
3000	43.6	45.4	47.5	49	52	55
3500	47.2	49.3	51.75	53.5	55.25	58.75
4000	49.8	51	53	55	57	61
4500	52.75	54.1	58.8	59.5	61.75	63.55
5000	55	57.5	63	63.5	70	73

**Fig. 5:**
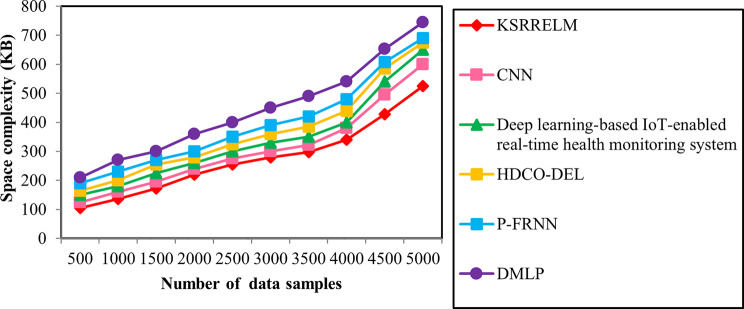
Graphical illustration of space complexity for lifesnaps Fitbit dataset

Figure 5 above depicts the space complexity of data classification using five different methods. The figure illustrates that the KSRRELM techniques minimized the space complexity of data classification compared to existing deep learning techniques. The overall result specifies that the time complexity of KSRRELM techniques is significantly minimized by 11, 19, 26, 32 and 40% compared to existing methods. This is because the KSRRELM technique performs a data preprocessing step where the outlier data is removed using Ratcliff-Obershelp pattern matching. This process efficiently minimizes space complexity.

**Fig. 6:**
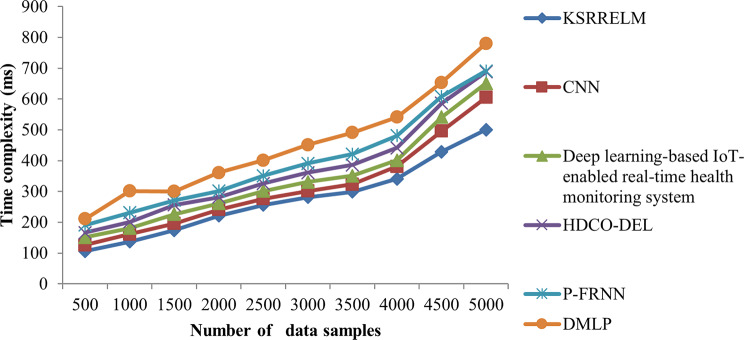
Graphical illustration of space complexity for MIMIC dataset

Figure 6 above depicts the space complexity of data classification using MIMIC dataset using five different methods. The figure illustrates that the KSRRELM techniques minimized the space complexity of data classification compared to existing deep learning techniques. The overall result specifies that the time complexity of KSRRELM techniques is significantly minimized by 11, 20, 26, 32 and 41% compared to existing methods. This is due to using proximity projection pursuit imputation method and Ratcliff-Obershelp pattern matching for minimizing the space complexity.

Table 6 illustrates precision, recall, and F1-score performance estimation using six methods, namely the proposed KSRRELM techniques and existing CNN [1] and a deep learning-based IoT-enabled real-time health monitoring system [2], HDCO-DEL [41], P-FRNN [4], and DMLP [36]. The figure illustrates that the KSRRELM techniques have higher precision, recall, and F1-score compared to existing deep learning techniques. This improvement is achieved by using recursive extreme learning machines. The overall performance of the precision using the KSRRELM technique is improved by 3, 6, 8, 11 and 2% when compared to [1, 2, 41, 4, 36], respectively. The overall performance of the recall using the KSRRELM technique is improved by 4, 5, 7, 9 and 2% when compared to [1, 2, 41, 4, 36], respectively. The overall performance of the recall using the KSRRELM technique is improved by 3, 6, 8, 10 and 2% when compared to [1, 2, 41, 4, 36], respectively.

**Table 6:** Comparison of precision, recall, and F1-score using Lifesnaps Fitbit dataset

Methods/Metrics	KSRRELM	CNN	Deep learning-based IoT-enabled real-time health monitoring system	HDCO-DEL	P-FRNN	DMLP
Precision (%)	94.42	91.86	89	87.22	85.21	92.42
Recall (%)	96.86	93.22	92	90.58	88.62	94.82
F1-score (%)	95.58	92.49	90.47	88.81	86.86	93.58

Table 7 illustrates precision, recall, and F1-score performance estimation using six methods, namely the proposed KSRRELM techniques and existing CNN [1] and a deep learning-based IoT-enabled real-time health monitoring system [2], HDCO-DEL [41], P-FRNN [4], and DMLP [36]. The figure illustrates that the KSRRELM techniques have higher precision, recall, and F1-score compared to existing deep learning techniques. This improvement is achieved by using recursive extreme learning machines. The overall performance of the precision using the KSRRELM technique is improved by 6, 9, 14, 11 and 5% when compared to [1, 2, 41, 4, 36], respectively. The overall performance of the recall using the KSRRELM technique is improved by 2, 4, 5, 8 and 3% when compared to [1, 2, 41, 4, 36], respectively. The overall performance of the recall using the KSRRELM technique is improved by 4, 7, 9, 11 and 3% when compared to [1, 2, 41, 4, 36], respectively.

**Table 7:** Comparison of precision, recall, and F1-score for MIMIC dataset

Methods/Metrics	KSRRELM	CNN	Deep learning-based IoT-enabled real-time health monitoring system	HDCO-DEL	P-FRNN	DMLP
Precision (%)	98.45	92.86	90	88.22	86.21	93.42
Recall (%)	96.52	94.22	93	91.58	89.62	94
F1-score (%)	97.58	93.49	91.47	89.81	87.86	94.58

**Fig. 7:**
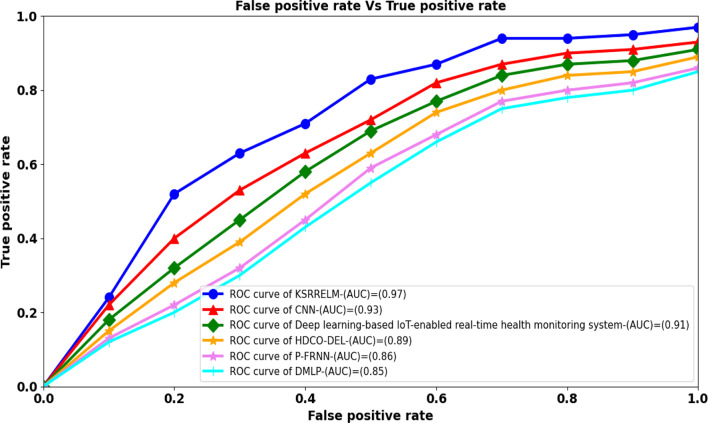
Graphical illustration of AUC

Figure 7 illustrates AUC performance estimation using six methods, namely the proposed KSRRELM technique and existing CNN [1] and a deep learning-based IoT-enabled real-time health monitoring system [2], HDCO-DEL [41], P-FRNN [4], and DMLP [36]. The AUC value ranges from 0 to 1. An AUC of 1 denotes a perfect model that accurately distinguishes between positive and negative classes, while an AUC of 0.5 suggests that the model’s performance is not efficient in prediction. AUC values less than 0.5 mean that the model’s performance is poor. In Fig. 7, the dotted straight line denotes a threshold point along the AUC curve, where the model’s performance is evaluated. Based on the observed results, the final AUC values are 0.96 for the proposed KSRRELM technique, 0.92 using [1], 0.9 using [2], 0.88 using [41], 0.85 using [4], and 0.84 using [36]. These results indicate that all models have very high prediction ability. However, the proposed KSRRELM technique outperforms the existing methods.

**Fig. 8:**
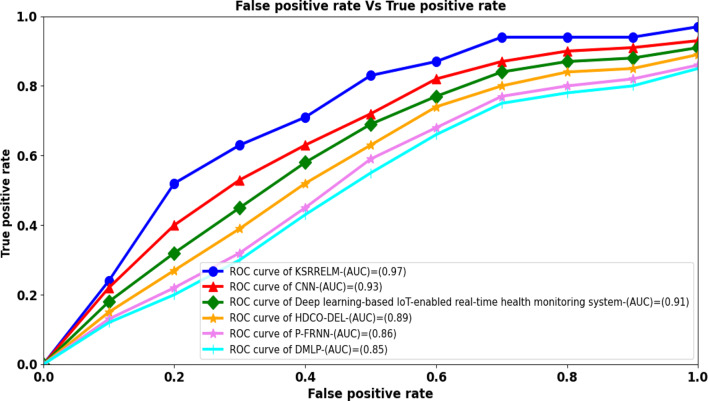
Graphical illustration of AUC

Figure 8 illustrates AUC performance estimation using MIMIC dataset of proposed KSRRELM technique and existing CNN [1] and a deep learning-based IoT-enabled real-time health monitoring system [2], HDCO-DEL [41], P-FRNN [4], and DMLP [36]. The AUC value ranges from 0 to 1. An AUC of 1 denotes a perfect model that accurately distinguishes between positive and negative classes, while an AUC of 0.5 suggests that the model’s performance is not efficient in prediction. AUC values less than 0.5 mean that the model’s performance is poor. In In Fig. 8, the dotted straight line denotes a threshold point along the AUC curve, where the model’s performance is evaluated. Based on the observed results, the final AUC values are 0.97 for the proposed KSRRELM technique, 0.93 using [1], 0.91 using [2], 0.89 using [41], 0.86 using [4], and 0.85 using [36]. These results indicate that all models have very high prediction ability. However, the proposed KSRRELM technique is better than the existing methods.

#### Statistical analysis

In our work, the statistical test for monitoring the health condition of patients is made by means of the **McNemar test** (Table 8). The McNemar test is considered a non-parametric testfor paired nominal data. In order to evaluate the McNemar test, the data are classified as normal or abnormal and placed into a 2 × 2 contingency table, with the cell frequencies equaling the number of pairs. Table 8 shows the McNemar test using proposed KSRRELM technique.

**Table 8:** Tabulation for the McNemar test for the proposed KSRRELM technique

	Wrongly predicted	Correctly predicted	
Wrongly predicted	8 (a)	14 (b)	22
Correctly predicted	4 (c)	474 (d)	478
	12	488	500

From the above table, b and c are utilized to measure the McNemar test statistics. The McNemar test formula is then measured as given below.

$${\chi ^2} = {{{{\left( {b - c} \right)}^2}} \over {b + c}}$$

**Fig. 9:**
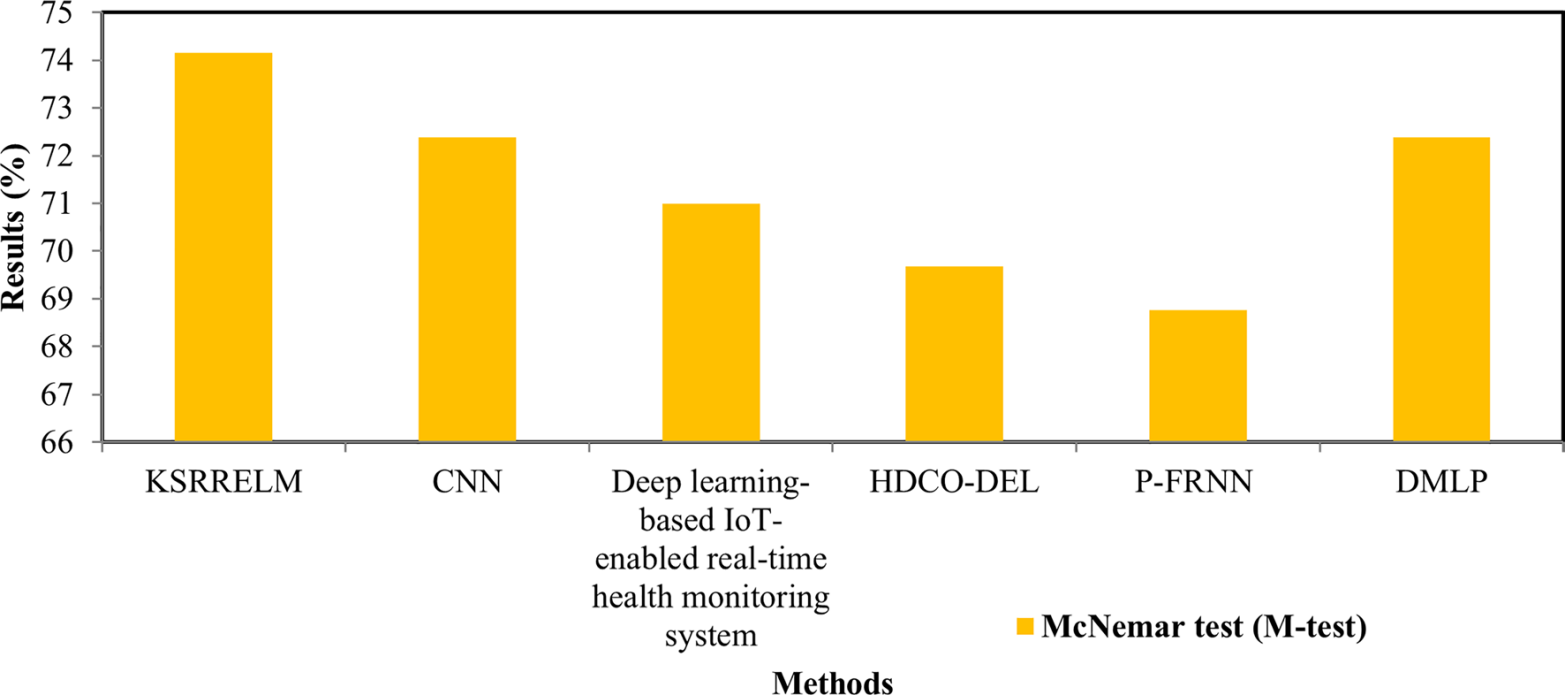
Results of McNemar test for Lifesnaps Fitbit dataset

Table 8 shows the tabulation for the McNemar test for the proposed KSRRELM technique. In a similar manner, with 500 to 5000 numbers of protein data points provided as input to the other six existing methods, the values are provided in Fig. 9.

Figure 9 demonstrates McNemar test performance estimation using six methods, namely the proposed KSRRELM technique and existing CNN [1] and a deep learning-based IoT-enabled real-time health monitoring system [2], HDCO-DEL [41], P-FRNN [4], and DMLP [36] for Lifesnaps Fitbit dataset. The overall performance of the McNemar test using the KSRRELM technique is improved by 2, 4, 6, 8 and 2% when compared to [1, 2, 41, 4, 36], respectively.

**Fig. 10:**
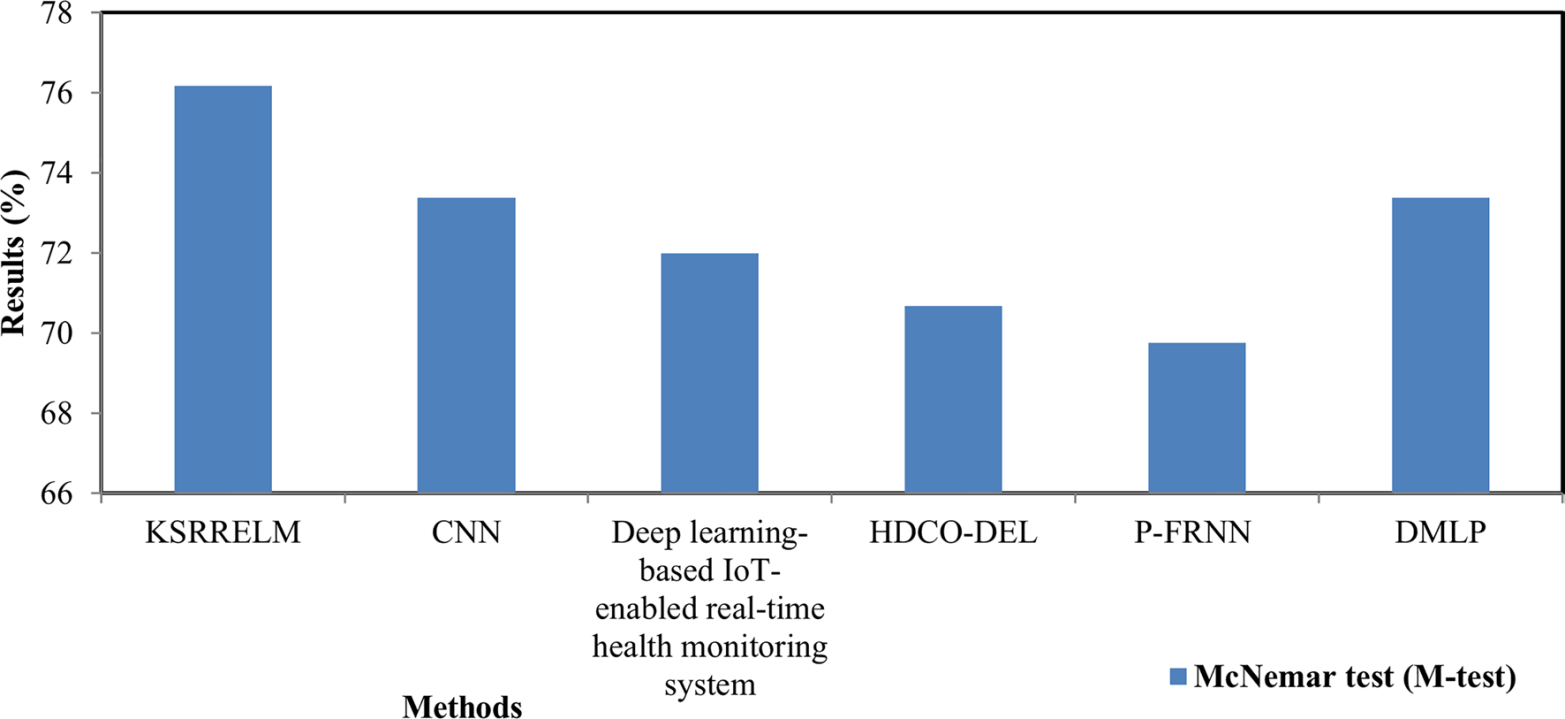
Results of McNemar test for MIMIC dataset

Figure 10 demonstrates McNemar test performance estimation using six methods, namely the proposed KSRRELM technique and existing CNN [1] and a deep learning-based IoT-enabled real-time health monitoring system [2], HDCO-DEL [41], P-FRNN [4], and DMLP [36] for MIMIC dataset. The overall performance of the McNemar test using the MIMIC dataset of the KSRRELM technique is improved by 4, 6, 8, 9 and 4% when compared to [1, 2, 41, 4, 36], respectively.

**Fig. 11:**
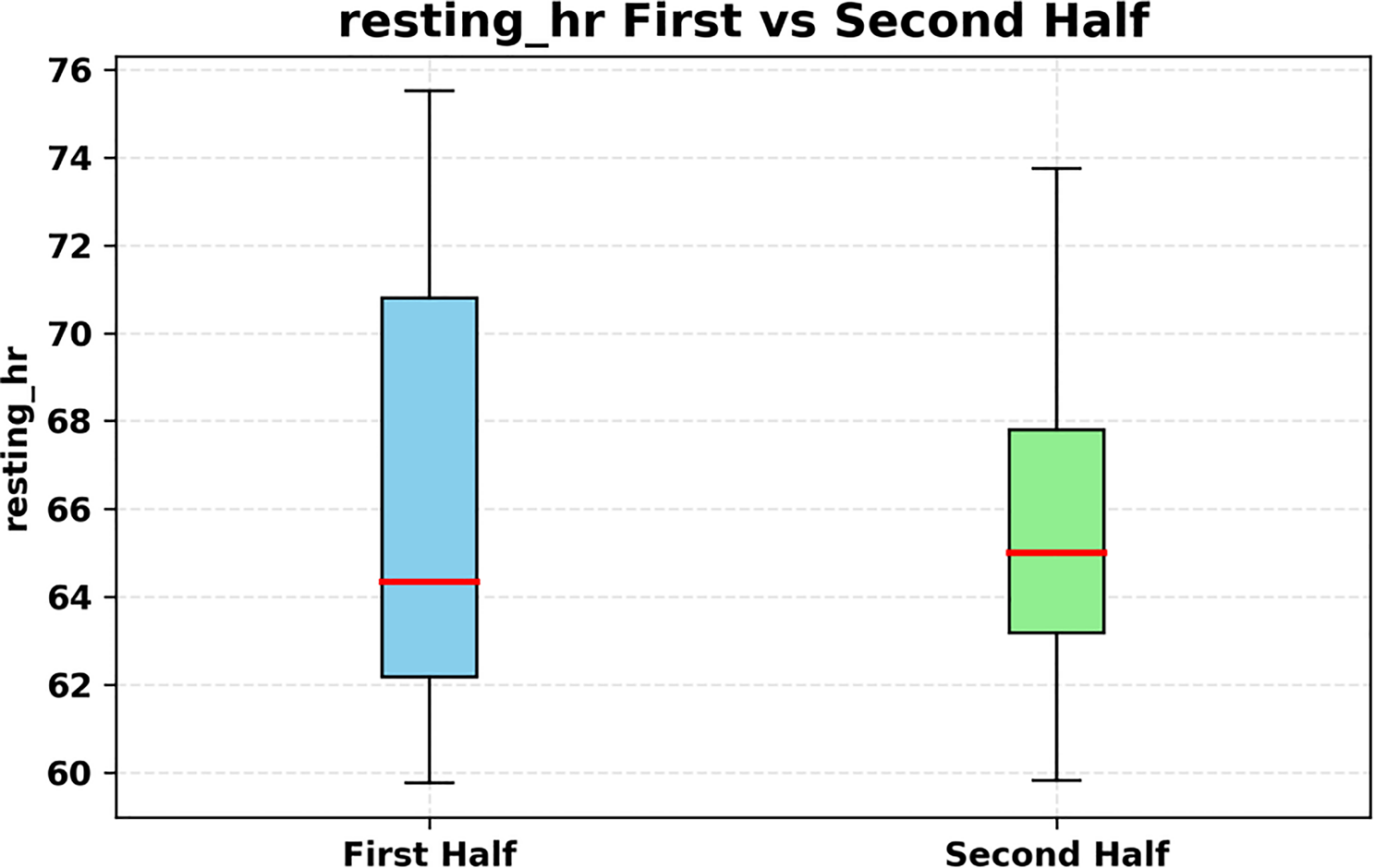
Wilcoxon signed rank

In our work, the statistical test for monitoring the health condition of patients is made by means of the Wilcoxon signed rank. Wilcoxon signed-rank test is a non-parametric statistical test employed to compare two related samples or to assess the median of a single sample against a hypothesized value. Figure 11 represents the Wilcoxon signed rank.

## Analysis sections

We conducted a case study on Apollo Group Hospital to provide clinical healthcare for patients located in remote areas. The KSRRELM technique helps provide remedial services to people living in distant areas. Apollo Health System implemented a remote patient monitoring system to track patients with physical and mental conditions remotely, including body temperature, oxygen saturation (SpO2), heart rate, beats per minute, calories, sleep duration, BMI, stress, etc. The system comprised wearable devices, mobile applications, and a centralized monitoring platform. Healthcare providers had access to this centralized platform to monitor patients’ health data in real-time. Figure 11 shows the case study diagram of the proposed KSRRELM technique.

**Fig. 12:**
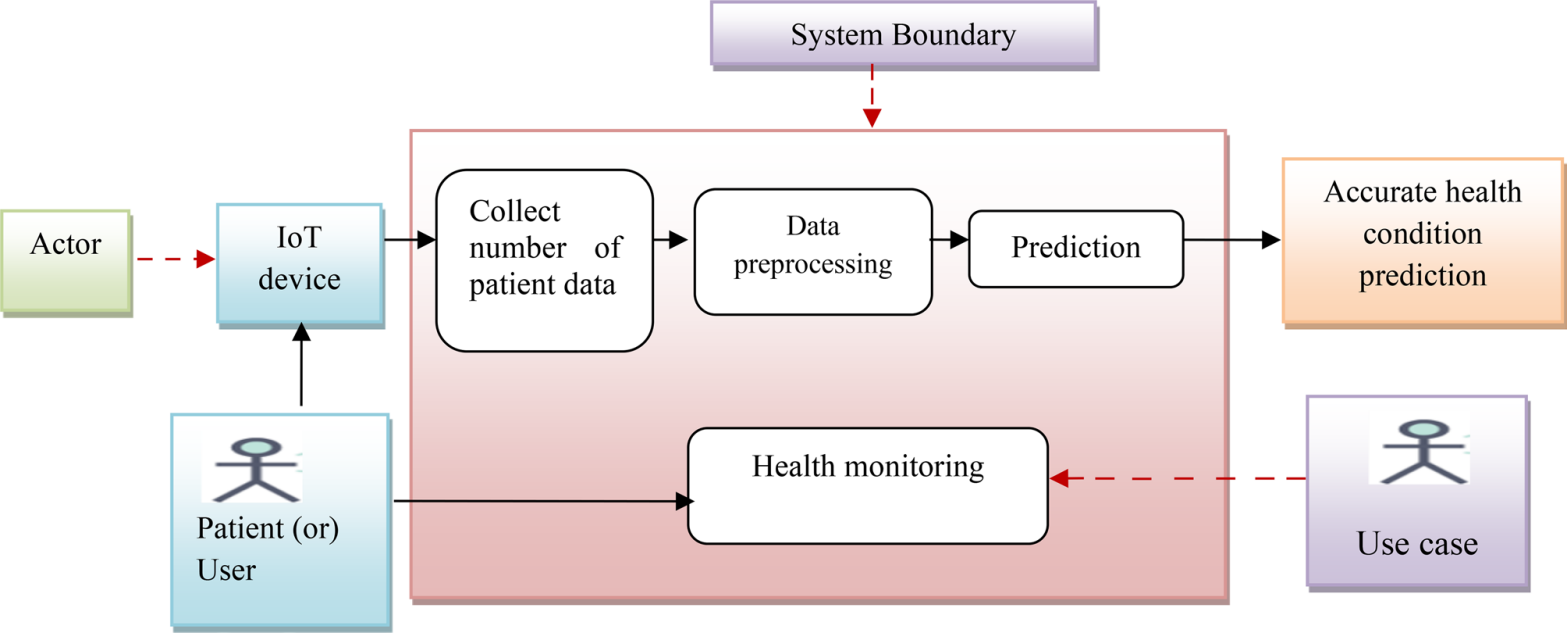
Case study diagram for health monitoring

Figure 12 presents the case study diagram of the proposed KSRRELM technique for accurate healthcare monitoring by using data acquisition, preprocessing, and prediction. An actor plays two roles, such as a doctor and a patient. A use case is a process of a system. The system boundary is defined in the needs of the document. The IoT devices are fixed on the patient for monitoring health. From the IoT devices, the number of patient data is collected. After collecting the data, the data preprocessing and prediction are employed in the proposed KSRRELM technique to achieve patient health conditions. This information is transmitted to the hospital server through the internet. As a result, the KSRRELM technique assists healthcare providers in generating automated alerts for critical patient conditions, enabling timely and accurate health condition prediction and reducing the risk of complications. The performance analysis of the KSRRELM technique revealed a significant improvement in disease prediction accuracy, with 8% increase and a reduction in error rate, time complexity, and space complexity by 56, 15, and 26% respectively, compared to conventional methods.

## Conclusion

Smart healthcare monitoring enables quick and efficient treatment or early detection of patient health issues, offering appropriate medications. This paper proposes an IoT-based KSRRELM technique for remote monitoring and early detection of health issues with higher accuracy and minimal time consumption. The KSRRELM technique collects healthcare information at different locations and time intervals. First, data preprocessing is performed to handle missing data and detect outliers, minimizing the time complexity of patient health conditions. Following this, kernel Slice Inversed Regression is applied in the recursive extreme learning machine to more accurately analyze patient physical and mental health conditions. Compared to the previous research, this proposed KSRRELM technique has several advantages, such as higher accuracy, precision, recall, and F1-score, and lesser time and space complexity. The quantitative performance results indicate that the presented KSRRELM technique has achieved accuracy as 95%, precision as 94%, recall as 97% and F1-score as 96%in health monitoring, along with a reduction in error rate as 5%, time complexity as 40 ms, and space complexity as 275 MB, when compared to existing methods. The limitation of the proposed KSRRELM technique is that it evaluates only one dataset and fails to perform feature selection; furthermore, only the minimum parameters are used. The limitations of the proposed work include data privacy and security, the need for large and high-quality datasets, the potential for algorithmic bias leading to inequities, and the high computational cost. In future work, the proposed technique is further extended for enhancing accurate health issues detection while minimizing the time by using novel preprocessing and feature selection methods to select relevant features and remove irrelevant features. Multimodal data fusion is used in future, Multimodal data fusion in health monitoring systems for providing more accurate, comprehensive, and reliable assessment of a patient’s health. Also, we will focus on more datasets for health monitoring to apply advanced deep learning with higher accuracy. Limitations in efficient health monitoring include high costs, data accuracy and misinterpretation, security and privacy vulnerabilities, patient fatigue from device charging and use, lack of interoperability between systems, internet connectivity issues, user training requirements, alarm fatigue for healthcare staff, and potential for minimized face-to-face patient-doctor interaction.

## Data Availability

The data is available within the article. The data have been gathered from [https://www.kaggle.com/datasets/skywescar/lifesnaps-fitbit-dataset] (https://www.kaggle.com/datasets/aakash941/mimic-dataset).

## Abbreviations

$Q$: Input of neurons
${\delta _{ih}}$: Weight between the input layer neuron and the hidden layer neuron
$$w$$: Bias
${X_i}$: Input patient data or training samples
$n$: Number of features or attributes
$m$: Number of patent data
$M$: Conditional statistical mean
$\sum {{X_i}}$: Sum of all the data values with the respective column
$P$: Outcome of proximity estimation
${X_{ij}}$: Neighboring data point
${X_m}$: Imputed data points
$$R$$: Matching coefficient
$\left| {{X_i}} \right|$ and $\left| {{X_j}} \right|$: Set’s cardinality and several terms in the given sets
$MD$: Mutual dependence between the data points
$$Z$$: Output function
$NDP$: Normal data point. If the coefficient is greater than 0.5
$ODP$: Outlier data point when the coefficient value is less than 0.5
$$\beta $$: Regression coefficient
$f$: Function relating the linear combinations of the input variables to the response variable $Y$
$K$: Gaussian kernel function that measures the relationship between the data ${X_i}$ and ${X_j}$
$H$: Hidden layer output
$A$: Sigmoid activation function
‘${\delta _{ho}}$’: Weight between the hidden layer neurons
$K$: Gaussian kernel function
$DF$: Output of the damped least-squares method
$\arg min$: Argument of minimum function
$$Y$$: Expected outcome
$Y{^{\prime}}$: Actual classification results
$$ERE$$: Error rate
${X_i}$: Number of data samples
$m$: Total number of data samples
$$TCY$$: Time complexity
$m$: Number of patient data or data samples
$time \left( {CDS} \right)$: Time for classifying the single data sample
$$SCM$$: Space complexity
$m$: Number of patient data or data samples
$MS \left( {CDS} \right)$: Memory space for classifying single-data samples
$$Pre$$: Precision
$${T_p}$$: True positive
$${F_p}$$: False positive
$${R_C}$$: Recall
$${F_n}$$: False negative
$$F$$: F1-Score
$${T_n}$$: True negative
